# The association between paternal housework and childcare with parents’ health in the postpartum period

**DOI:** 10.1016/j.midw.2026.104777

**Published:** 2026-03-13

**Authors:** Chelsea L Kracht, Chris M Blachard, Danielle Symons Downs, Mark R. Beauchamp, Ryan E. Rhodes

**Affiliations:** a University of Kansas Medical Center, Kansas City, KS, USA; b Dalhousie University, Faculty of Medicine, Halifax, Canada; c The Pennsylvania State University, Departments of Kinesiology and Obstetrics and Gynecology, University Park, USA; d The University of British Columbia, School of Kinesiology, Canada; e University of Victoria, School of Exercise Science, Physical and Health Education, Victoria, Canada

**Keywords:** Postpartum, Father involvement, Exercise

## Abstract

**Problem:**

The postpartum period is associated with poor maternal mental and physical health.

**Background:**

Parental domestic labor may help support maternal health; little is known about how it may help during the postpartum period.

**Study aims:**

1) describe the trend of father’s domestic labor (childcare and housework) across the postpartum period, 2) examine the agreement between perceived and reported domestic labor between mothers and fathers, and 3) explore the relationship between father’s domestic labor and parental outcomes.

**Methods:**

These secondary analyses included fathers and mothers from a 6-month longitudinal randomized controlled trial to increase new parents’ physical activity (PA) in the postpartum period. Fathers and mothers self-reported their domestic labor (childcare and housework, levels: low [1–25 %], middle [26–50 %] or high [51–75 %]), perceptions of partner’s domestic labor, PA, sleep, stress, and physical health at 2-months, 4-months, 6-months, and 8-months postpartum.

**Findings:**

Among 164 individuals (52 intact couples, 31.5 ± 4.5 y, 53.6 % mothers, 82.8 % White), fathers self-reported they contributed a middle amount of childcare across time, and initially a high amount of housework (2-months) which was reduced to the middle amount at other time points. Mothers’ perception was positively related to fathers’ domestic labor. Fathers’ perception was positively related to mothers’ housework. Fathers were divided into three domestic labor involvement categories; these categories were associated with higher maternal PA.

**Discussion:**

These findings highlight the fathers’ contribution to domestic labor in the postpartum period, and opportunities to support parental health behaviors.

**Conclusions:**

Interventional evidence is required to optimize domestic labor and new parent’s health.

## Introduction

The first year of postpartum is a significant transition for all parents. This transition is well documented in mothers, including changes in their physical, psychological, and social roles, which impact their own physical and mental health (Neshteruk et al., 2022). This pivotal period has recently been recognized with multi-behavior guidelines to support maternal health (Dipietro et al., 2019; Evenson et al., 2024; Lane--Cordova et al., 2022), such as the Canadian guidelines for physical activity (PA), sedentary behavior, and sleep (Davenport et al., 2025). The Canadian guidelines propose that mothers beginning or returning to activity should achieve 120 min/week of moderate-to-vigorous physical activity [MVPA] early in the postpartum period, along with adequate sleep (e.g., 7–9 h/night) and good sleep hygiene (e.g., avoid screen-time before bed) to support their mental health and reduce stress (Davenport et al., 2025). Still, the postpartum period is typically characterized by inadequate sleep behaviors (duration, awakenings, etc.) and difficulty resuming PA for the mother and, to a lesser extent, the father (Bellows-Riecken and Rhodes, 2008; Le Bas et al., 2025; Nielsen et al., 2025). These behavior changes result in poor mental health, such as postpartum depression (Divine et al., 2022), which can also negatively impact a child’s growth and development through lower rates of breastfeeding and less infant bonding with the mother (Slomian et al., 2019). Efforts to support adequate parent health behaviors, and physical and mental health may have long-term benefits for the parents and children.

Social and childcare support can improve the mother’s health behaviors, reduce symptoms of postpartum depression and anxiety (Song et al., 2024; Zhang et al., 2023), and serve as key avenues for fathers to contribute to parent and child health in the first year postpartum. Father involvement may also lead to long-term child mental health benefits, including improved psychological well-being in childhood and adolescence (Kato et al., 2023). Specific to the postpartum period, existing evidence documents that fathers’ involvement in childcare postpartum is protective against maternal postpartum depression and anxiety (Lin et al., 2017; Zhang et al., 2023; Zhang and Razza, 2022), potentially through mechanisms of mitigating the adverse effects of short sleep on maternal health and increasing maternal sleep (Armstrong et al., 2022). Indeed, inadequate maternal sleep is a well-known correlate of postpartum depression (Owais et al., 2018), likely from additional childcare time during the day and night. Maternal sleep disturbances may also be mitigated by father involvement in childcare (Armstrong et al., 2022; Song et al., 2024). For example, a longitudinal study of 666 mothers found that when mothers had help with childcare (e.g., family members), there was no link between short sleep duration and depression (Armstrong et al., 2022). However, when mothers did not have help with childcare, short sleep duration was associated with higher depressive symptoms (Armstrong et al., 2022). Another cross-sectional study of 290 mothers demonstrated that higher paternal involvement in nighttime childcare was directly linked to lower maternal insomnia characteristics (Song et al., 2024).

Despite some evidence for the role of childcare and household support in maternal health behaviors and parental mental and physical health postpartum, current evidence is limited with respect to five main factors. These include 1) evidence based predominantly on cross-sectional studies (deMontigny et al., 2020; Lin et al., 2017; Song et al., 2024; Zhang et al., 2023), 2) involvement duties among parents are not well defined in such studies (Armstrong et al., 2022; Suzuki et al., 2022), 3) failing to consider the mother’s perception of paternal involvement in duties (deMontigny et al., 2020; Zhang et al., 2023), 4) not considering differing family structures (e.g., multiple children and single child), and 5) primarily conducted in Japan or China (Song et al., 2024; Zhang et al., 2023), limiting wider relevance. Prospective studies may help identify changes in childcare and housework and inform policymakers on the impact of paternity leave length on parents’ overall health (Heshmati et al., 2023). Mothers who feel they are not supported in domestic labor (e.g., childcare and housework) may experience depleted relationship satisfaction and higher conflict (deMontigny et al., 2020), with negative implications for both parents and child health. Moreover, having additional children in the home may impact the amount of housework and childcare, as parents with multiple children may perform more housework relative to those with one child (Divine et al., 2021). Finally, current evidence is primarily focused on Asian countries limiting relevance to North America which may have differences in parenting practices and cultural expectations for domestic labor (Schindler et al., 2025).

The current study builds upon past investigations and these current gaps in the evidence by examining paternal involvement multiple times across the postpartum period, capturing both housework and childcare, and mothers' perceptions of fathers' contributions in a group of new parents (mothers and fathers). Through three aims, this study seeks to characterize paternal involvement across the postpartum period. The first aim tests the hypothesis that fathers will contribute more to childcare and housework while they are on leave. The second aim is to examine agreement with mothers’ perception of the father’s involvement in domestic labor and father’s reported involvement in domestic labor to test the hypothesis that mothers will perceive fathers as completing less labor. The final aim examines the contribution of paternal domestic labor on father and mothers’ health behaviors and mental and physical health, with the hypothesis that increased father involvement in domestic labor will reduce maternal stress and improve maternal sleep. These hypotheses were developed based on previous literature in father involvement and maternal health (Armstrong et al., 2022; deMontigny et al., 2020; Lin et al., 2017; Song et al., 2024; Suzuki et al., 2022; Zhang et al., 2023).

## Methods

### Participants and procedure

This report is a secondary analysis of data derived from a two-arm parallel randomized trial that sought to evaluate the extent to which a parenting intervention improved MVPA during the postpartum period in couples expecting their first child who resided in a city of a North American province. This 6-month study occurred between 2014–2018 (Rhodes, Blanchard, et al., 2021). At that time, most working couples had 15-weeks of paid leave after birth, plus 35 shareable weeks between mothers and fathers, with some mothers returning to work at 8-months postpartum (Baker and Milligan, 2008; Canada, 2024). Based on national estimates, around 30 % of partners utilized the shareable weeks in 2014–2018 (Canada, 2024). During this period, partnered mothers did 60 % of unpaid housework, likely due to societal expectations (Sinclair and Besporstov, 2022; Thébaud et al., 2019). This study was conducted prior to the 2019 changes of “use it or lose it” legislation for the shareable benefit in this country (Canada, 2024).

The associated university’s Research Ethics Board approved the study (reference #11–036), and it was registered with the Clinical Trials Registry (NCT02290808). The main trial results found that fathers' MVPA was stable in both conditions (i.e. intervention and control), but the mother’s MVPA increased six weeks into the trial but either declined (control participants) or remained steady (intervention participants) (Rhodes, Blanchard, et al., 2021). Primary outcome results and full study details are published elsewhere (Quinlan et al., 2017; Rhodes, Beauchamp, et al., 2021). Based on the number of measurement occasions, 6-month duration, a within-person variance of 1.0, potential growth rate of 1.0, and a moderate effect size (0.4), the total number of participants needed to show a significant adherence to MVPA recommendations was 200 individuals (i.e., 100 couples/condition) (Tremblay et al., 2011). This amount was increased to 267 individuals to account for a potential 25 % attrition rate based on another longitudinal study (Rhodes et al., 2014). In total, 264 participants completed the main study, and 164 participants provided complete data at all time-points for this secondary analysis study.

Common-law or married couples who were expecting their first child and were >18 years of age were included. Couples were excluded if they were not ready to engage in PA as measured by the Physical Activity Readiness Questionnaire for Everyone (Warburton et al., 2011). Couples were not excluded for depression or anxiety, since they are small contraindications for PA (Rhodes et al., 2011). Prenatal PA participation was not an exclusion criterion, as parents may no longer be active postpartum (Rhodes et al., 2014). Further, the intervention focused on preventing PA decline and improving PA (Rhodes and Quinlan, 2015). Sampling methodologies included convenience and snowball sampling, Recruitment methods included study announcements and/or paper advertisements at online interest sites, local community events, clinical avenues, midwiferies, and recreation centers. In addition, enrolled participants were invited to refer other families to participate in the study.

Baseline measurements were conducted at ~2-months postpartum. Before inclusion was confirmed and baseline data were collected, informed consent was obtained in writing. At baseline, participants completed in-person questionnaires related to childcare, housework, health behaviors, stress, physical health, a fitness test, and wore an accelerometer for 7-days to assess PA. Parents reported whether they were on maternity leave (yes or no), their partner was on leave (yes or no), and mothers reported whether they were breastfeeding (yes or no). After baseline measures, participants were randomized in a 1:1 ratio to either the intervention or the control group using an online randomization program (Quinlan et al., 2017). The subsequent two study visits (4-months and 6-months postpartum) involved completing questionnaires via email using SurveyMonkey (Ottawa, ON, Canada) and accelerometry (over the ensuing 7-days) and corresponded with intervention booster sessions. At the final study visit (8-months postpartum), participants repeated questionnaires in-person, accelerometry (as at pre-test and the two prior study visits), a fitness test, and end-of-study surveys. Accelerometry adherence was not used as a primary measure for movement behaviors since missing data was not at random (Rhodes, Beauchamp, et al., 2021). Rather, the current analysis utilized self-report measures of movement behaviors. All variables included in the analysis were assessed at each of the four visits.

### Measures

#### Housework and childcare

Parents were queried about their childcare and household responsibilities during the past week. Specifically, they were asked “*during the last week, how often did you contribute towards work around the house?”* Similarly, fathers and mothers were asked two separate questions about how often their partner contributed towards work around the house. These questions had the same response options. As for childcare, parents were asked “*during the last week, how often did you help with the childcare of your child*?” This question was repeated for their partner. Housework and childcare questions had the same response options of none (0 %), a little (1–25 %), some (26–50 %), a fair bit (51–75 %), most (75–100 %), or all (100 %).

#### Sleep and physical activity

Sleep duration was assessed using a question from the Pittsburgh Sleep Quality Index (Buysse et al., 1989). Participants were asked to report the number of hours of *actual* night-time sleep over the past month in 0.5-hour increments.

PA was operationalized as MVPA and was assessed using the Godin-Shepard Leisure-Time Questionnaire, which inquiries about weekly frequency and duration of varying PA intensities. This questionnaire was modified to include an open response option of bout duration (Courneya et al., 2004; Godin et al., 1986; Godin and Shephard, 1985). In three separate questions, participants were asked over a typical week the average frequency and duration they spent in mild (or light PA), moderate PA, and vigorous PA. The average frequency was multiplied by the average duration to calculate the average exercise minutes per PA intensity. Average moderate and vigorous PA minutes were summed to calculate MVPA minutes/week based on previous analyses (Courneya et al., 2004).

#### Stress

Stress was measured using the Perceived Stress Scale questionnaire, which addresses 10 feelings and thoughts over the last month (Cohen et al., 1983). Among 10 questions, participants were asked to indicate their global stress level and the frequency of with which they experienced stress. Questions had five response options (score: 0–4), ranging from “never” to “very often” and were reverse coded for four questions. An example question is “*in the last month, how often have you found that you could not cope with all the things that you had to do?*”. Responses were summed to yield a total score (range: 0–40), with higher scores indicating greater stress. Reliability was evident from scores derived by this instrument with a Cronbach α values of 0.89, 0.87, 0.87, 0.87, for time 1–4, respectively. Internal consistency values are similar to other prospective studies of new parents’ behavioral control (Divine et al., 2021) and stress across the perinatal period (Benediktsson et al., 2017).

#### Physical health

Physical health was measured via the Physical Quality of Life (PQoL) scale from the Short Form-12 Part of the Quality of Life Survey (Ware et al., 1996). From the 12-question survey, six questions referenced their current health (or “now”) or within the past week, with questions specific to physical functioning, physical functioning in role, bodily pain, and general health. These questions had 3–5-point Likert scale responses and were summed to obtain a final score, which ranged from 6 to 28, with a higher score indicating a greater PQoL. In the current analysis, instrument-derived scores demonstrated Cronbach α values of 0.87, 0.87, 0.86, 0.89 for time 1–4, respectively. These values are consistent with the initial test-re-test reliability (Ware et al., 1996) and another postpartum examination (Hirose et al., 2020).

### Statistical analysis

Individuals with complete data for age, sex, childcare, housework, mental and physical health, and sleep were included. Normality was investigated for dependent variables, and non-normally distributed values were presented as median with interquartile ranges. For the first aim, to describe father involvement in domestic labor across the postpartum period, central tendencies were calculated for fathers’ housework and childcare across each time point. Few fathers (<5 %) reported none (0 %), most or all (75–100 %), or all (100 %) for domestic labor categories (see Supplementary Figure 1). Thus, fathers reporting the response of none (0 %) collapsed into a little (1–25 %), and fathers reporting most or all (75–100 %), or all (100 %) collapsed with a fair bit (51–75 %). The final categories were low (A little 1–25 %), middle (some, 26–50 %), or high (A fair bit, 51–75 %).

For aim 1, a mixed effects model was used to examine changes over time in childcare and housework. Models were conducted separately for mother’s perception and father self-reports, with all available data included in each model, respectively. We further examined the relationship between housework and childcare at each time controlling for time point specific maternity leave, partner leave, sleep, MVPA, stress, and PQoL using chi-square for categorical variables and analysis of variance for continuous variables. Aim 1 was conducted with all available data, thus uncoupled individuals could be included in analysis. Aim 2 and 3 included couple data. For aim 2, a linear mixed model was conducted to examine the relationship between the parents’ reported contributions across time and the partner’s perception of childcare contributions, with separate models for mothers and fathers. This model included the main effect (perception), time, and time*perception interaction, and the outcome of partners’ reported contribution. For aim 3, to examine how fathers’ domestic labor affects parents’ health, fathers were grouped into three equal categories based on their contributions to housework and childcare. These groups were created by analyzing patterns and overlaps in fathers’ reported housework and childcare, ensuring each group was a similar size (see Supplementary Table 1). These categories were used as independent variables in mixed effects models that examined the effect of group and time interaction on the dependent variables of fathers’ and mothers’ sleep, MVPA, stress and PQoL, in separate models. Models were adjusted for potential time-varying covariates determined *a priori*, including their maternity or paternity leave status, mother’s nursing status, their sleep duration, and partner’s perception of housework and childcare based on existing literature (Armstrong et al., 2022; deMontigny et al., 2020; Suzuki et al., 2022). Analyses were conducted in SAS 9.4 (Cary, N.C.), and significance was set at *p* < 0.05.

## Results

One hundred and sixty-four individuals were included in the main analysis for this study, composing 52 intact couples (both partners), and 60 independent parents that contributed complete data. As shown in Table 1, participants were 31.5 ± 4.5y, majority were White (82.8 %), held a college or university degree (50.9 %) or a professional or graduate degree (31.2 %) and most mothers (*n* = 88) reported breastfeeding across time (82.5–94.3 %).

Differences in independent variables (childcare, housework, and perception of partner), dependent variables (sleep, MVPA, stress, and PQoL), or key confounders (age, sex, leave status, and breastfeeding) were minimal between individuals included and excluded from the analysis. Individuals not included in the analysis (*n* = 100) reported lower PQoL (4-months, median[IQR]: 18[16,19], 8-months: 18[16,19]) compared to individuals (*n* = 164) who were included (4-months: 18 [17,19], 8-months: 18[17,19], *p* < 0.01 for both). Furthermore, individuals not included were more likely to have vocational education or some college experience compared to individuals included in the analysis (9.8 %, *p* = 0.01).

### Aim 1: characterize paternal involvement across the postpartum period

#### Housework

**Fig. 1.**
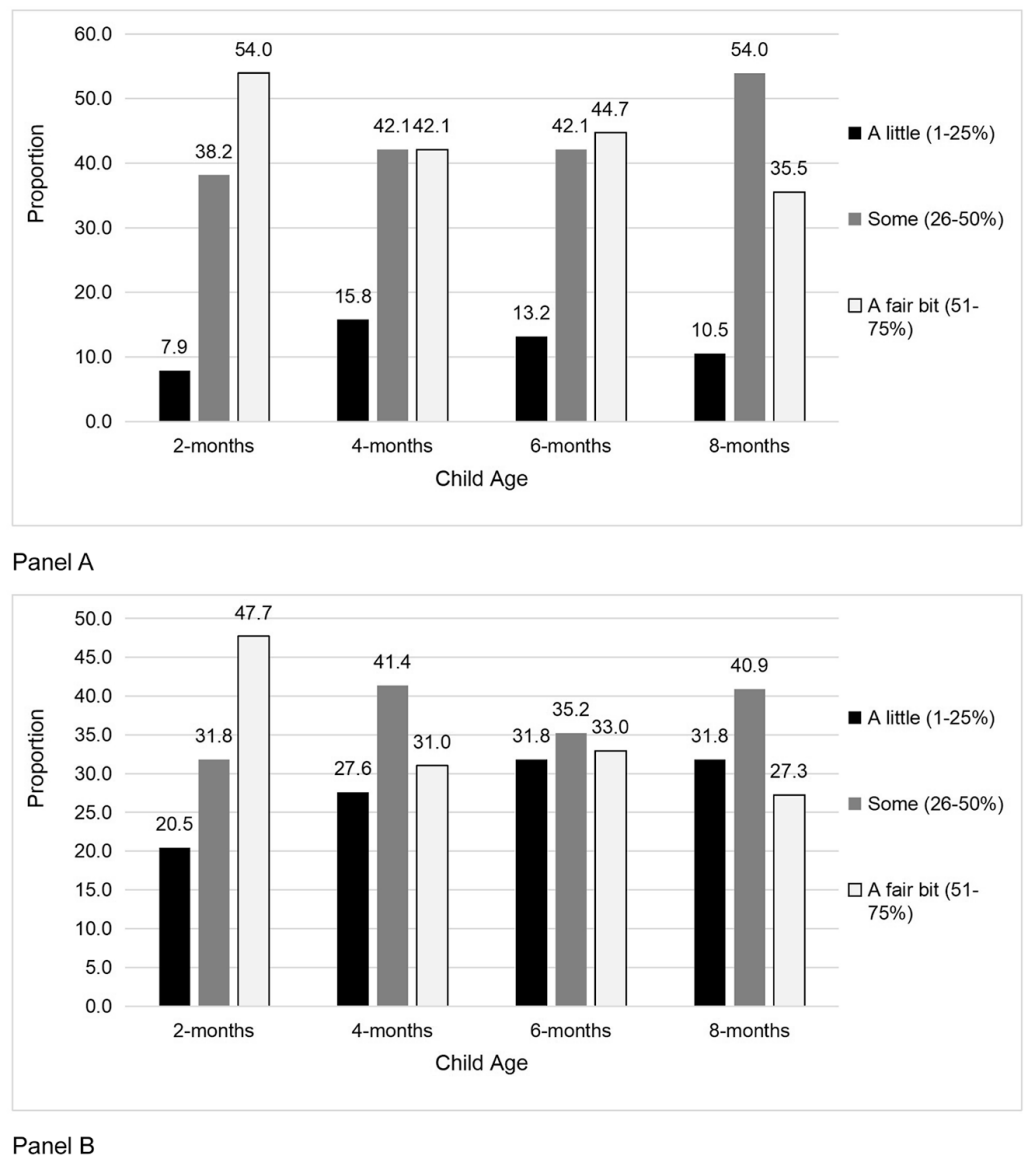
Distribution of Father's Reported and Mother’s Perception of their Housework Contribution across time. Panel A: Father’s Reported Housework Contribution; Panel B: Mother's Perception of Father's Housework.

Fifty-four percent of fathers were in the high housework category (i. e., a fair bit, 51–75 %) at 2-months but reverted to the middle category (i.e., some, 26–50 %) for the subsequent periods (range: 42.1–54.0 %, Fig. 1a); the fathers’ housework contribution did not change across time (*p* = 0.07). Mothers' perceptions generally aligned with those of fathers but changed over time (*p* = 0.01). Mothers perceived slightly more fathers in the low housework category (i.e., a little, 1–25 %) across time (20.5–31.8 %, Fig. 1b). At 2-months, there was a significant difference in housework category by paternity leave (*p* = 0.04). Fathers who were not on leave were primarily in the high category (7/8 individuals, 87.5 %), while those on leave were primarily in the middle (29/68 individuals, 42.6 %) and high categories (34/68 individuals, 50.0 %). At 2-months, father’s stress differed by housework category (*p* = 0.04), but no significant pair-wise comparisons were found. There were no other significant differences between fathers’ housework contributions (*p*’s > 0.05).

#### Child care

**Fig. 2.**
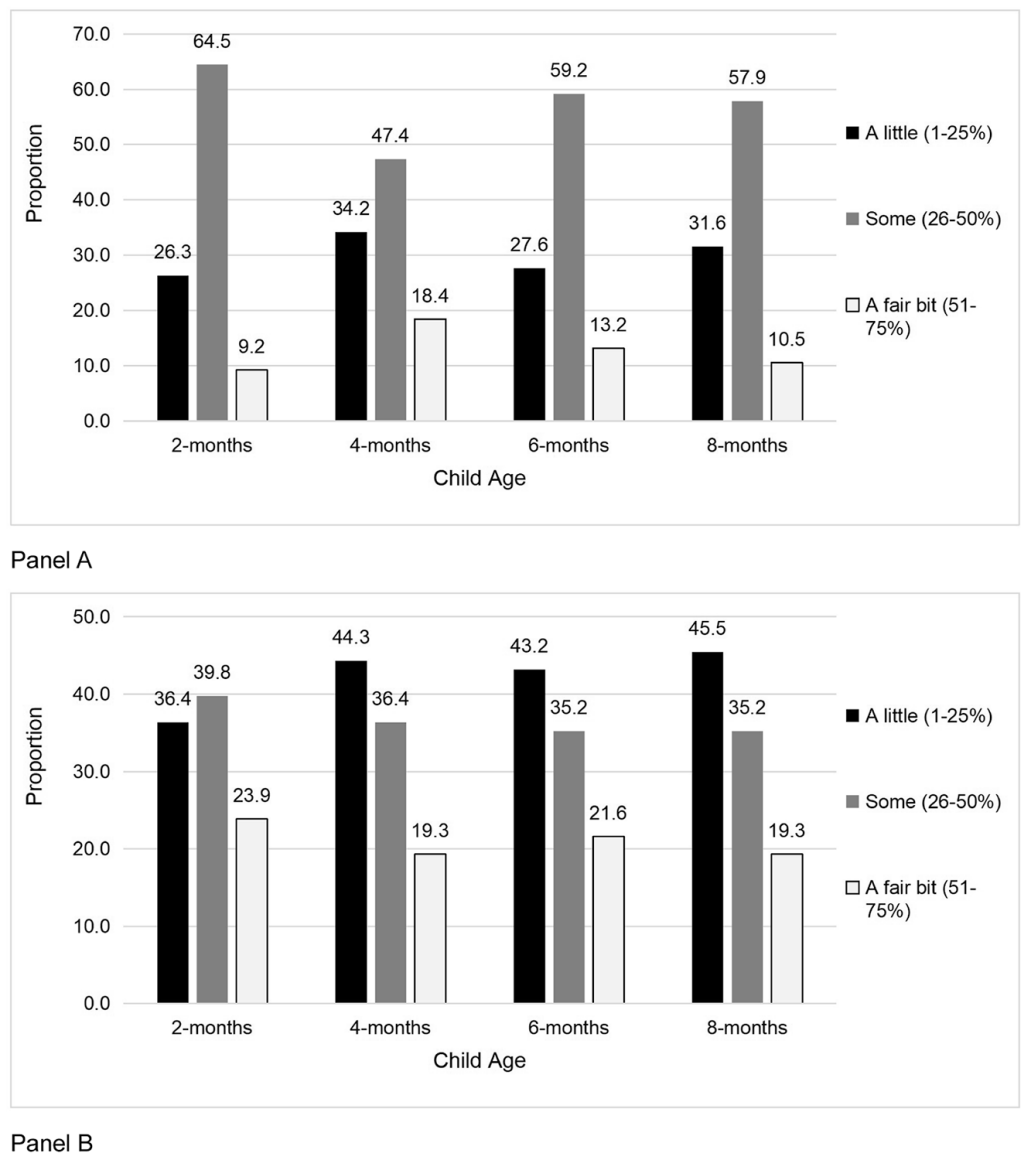
Distribution of Father's Reported and Mother’s Perception of their Childcare Contribution across time. Panel A: Father’s Reported Childcare Contribution; Panel B: Mother's Perception of Father's Childcare.

As shown in Fig. 2a., the majority (48.4–64.5 %) of fathers were in the middle childcare category across time. These results somewhat align with the mother’s perception, as more mothers reported fathers in the middle category (range: 39.8–45.5 %, Fig. 2b). These amounts were stable across time for fathers’ reporting (*p* = 0.40) and mothers’ perception (*p* = 0.87).

Father’s childcare contribution differed by their paternity leave at 8-months (*p* = 0.01). At 8-months, fathers who were on leave reported in the middle (3/6, 50 %) or high category (3/6, 50 %), whereas fathers who were not on leave were represented across all childcare categories. Similarly, at 4-months (*p* = 0.02), 6-months (*p* < 0.01), and 8-months (*p* = 0.01), fathers without a partner on leave reported the middle or high childcare category, compared to fathers with a partner on leave. Still, most mothers were on leave between 4–8 months (76–86 %).

At 4-months postpartum, fathers in the low childcare category had higher stress scores (15.9 ± 5.92) than fathers in the middle category (11.5 ± 5.64, *p* = 0.01). There were no other significant differences between fathers’ childcare contributions (*p*’s > 0.05).

### Aim 2: agreement in housework and childcare between mothers and fathers

In separate models, mothers’ perception was positively related to fathers’ reported housework (*p* < 0.01) and childcare contribution (*p* < 0.01) and this did not change across time (*p*’s > 0.05). Fathers’ perception was positively related to mothers’ reported housework contribution (*p* = 0.03), but not childcare (*p* = 0.64). Notably, mothers and fathers agreed that the mother performed most of the childcare at 2-months, so no estimates were able to be calculated for that time point.

### Aim 3: father’s housework and childcare on couple’s health behaviors, stress, and physical health

There was a significant difference in childcare and housework distribution at 2-months, 4-months, and 6-months. Using 2-month data, we created three groups 1) lower domestic labor category including low childcare and low, middle, or high housework (*n* = 20, herein: lower category), 2) middle domestic labor category including middle childcare – low or middle housework (*n* = 21, herein: middle category), and 3) high domestic labor category including middle or high childcare and high housework (*n* = 35, herein: high category). This distribution is mirrored within those with complete couples’ data (see Supplementary Table 1). Contrasting individual examinations, in adjusted models, there was no significant group, time or interactions for fathers’ stress. As shown in Table 2, fathers in the lowest domestic labor category initially experienced high stress but their stress decreased. Fathers in the middle domestic labor category initially experienced mild stress which increased across time. The high domestic labor category experienced some stress across time but ended with the highest stress score. There were no other significant associations between group, group*time, with father’s outcomes.

As for maternal outcomes, the group variable was related to mothers’ MVPA (*p* = 0.04, Table 2). Mothers with partners in the middle domestic labor category achieving high MVPA amounts at 2-months likely drove this result; the mothers with partners in the high domestic labor category slightly increased their MVPA across time, while the mothers with partners in middle domestic labor category gradually decreased their MVPA across time. There were no other significant associations between group or group*time interaction with mothers' stress, QoL, or sleep (*p*’s > 0.05). In all non-sleep models, maternal sleep was a significant contributing factor (MVPA model, Estimate ± SE: 16.5 ± 9.7, *p* = 0.08; PQoL: 0.19 ± 0.09, *p* = 0.03; Stress: −1.09 ± 0.32, *p* < 0.01).

## Discussion

The purpose of this study was to utilize data derived from an PA promotion trial to describe paternal involvement across the postpartum period, examine agreement between mother’s perceptions and father’s report of domestic labor, and understand the contribution of paternal domestic labor on parent’s health behaviors. The current investigation improved upon past literature by including a detailed assessment of both childcare and housework in fathers, capturing both parent’s perceptions and reports, and a longitudinal assessment during the postpartum period. Fathers reported a moderate contribution to childcare and housework over time. Mothers perceived that fathers contributed slightly less to domestic labor but were generally in agreement with fathers about fathers’ domestic labor contributions. Fathers experienced high stress, and mothers were active at various periods. These results highlight that fathers’ domestic labor may not reduce the high stress of the postpartum period but may have benefits for the mother’s health behaviors, with positive implications for her later physical and mental health. Furthermore, these findings support parent-based programs to promote PA and reduce parental stress for benefiting parent health.

We found some support for our hypothesis that fathers would contribute to more domestic labor while on leave. In this sample, fathers on leave reported slightly more involvement (middle or high category), relative to those not on leave; those not on leave were represented in all categories (low to high). Many fathers were on leave for the first 6-months which limited comparisons with fathers not on leave (*n* = 6–8/period). High utilization of leave is a positive result in itself (Heshmati et al., 2023), and may reflect the North American context (Heshmati et al., 2023). Still, fathers who take longer leave have lower paternal stress and anxiety relative to those who take no leave at all (Cardenas et al., 2021). Fathers contributed slightly more to housework relative to childcare; this amount was moderate and stable across time. The stable father housework contributions align with another longitudinal investigation of housework across varying types of couples (non-parents, new parents, and established parents) (Divine et al., 2021). In that study, fathers reported most of their housework was meal preparation and cleaning, which are ongoing tasks needed across the postpartum period.(Divine et al., 2021). Further assessment of father’s domestic labor across differing leave statuses (i.e., those on leave and not leave) may improve upon these findings of a predominately leave-taking group.

We found support for our hypothesis that mothers would perceive fathers completing less overall domestic labor. The mothers’ perception of the fathers’ domestic labor proportions is slightly lower than another postpartum study conducted in China. In that study, mothers reported that fathers “often” performed such childcare tasks as feeding and hygiene, playing and reading, and involvement when the child is sick (Zhang et al., 2023). That study inquired near the end of the postpartum period (~1 year), rather than individual timepoints across time (Zhang et al., 2023). In the current study, all parents agreed mothers performed most of the childcare responsibilities at 2-months. Though agreement may have positive indications for couple’s relationship quality, relative to disagreements or unmet needs (Biehle and Mickelson, 2012), over-burdening the mother with childcare responsibilities may have independent effects for her mental health (Song et al., 2024; Zhang et al., 2023). Opportunities for fathers to support mothers in the early postpartum period by contributing to additional childcare may have positive implications for her later mental health.

We did not find support for the hypothesis that high paternal involvement reduced maternal stress or improved maternal sleep. Rather, our results indicate varying father stress and maternal MVPA paths based on fathers’ initial childcare contributions. The current study created groups based on 2-month domestic labor categories, and these initial domestic labor contributions may not generalize contributions at 8-months or later postpartum, and specific time points (e.g., return to work) may be more meaningful than others. The 6-month period has been noted as a critical time for health promotion in fathers due to the fathers obtaining shortened sleep for an extended period (Lo et al., 2021) and potentially fathers returning to work. In this sample, the shifts in father stress later in the postpartum period may be tied to utilization of shared leave. The higher stress at 4-months may correspond to returning to work after 15-weeks of paid leave (Canada, 2024). The high amount of stress at 8-months may correspond to transitions in the home, such as a realigning of duties as the mother returns to work (Baker and Milligan, 2008; Canada, 2024). Nevertheless, all fathers exhibited higher stress levels (>14.00) at some points; however, this amount is normative for males (Mean ± SD: 14.46 ± 7.81) and those with higher household incomes (Mean ± SD: 14.74 ± 6.88) (Cohen and Janicki-Deverts, 2012). As for mothers, those with partners contributing a middle amount of domestic labor were meeting the Canadian guidelines (120 min/week) at 2-months (Davenport et al., 2025); other groups achieved this amount in later time periods. It is unclear why mothers who have a partner contributing a middle amount of domestic labor, rather than a high amount, obtained more MVPA. The models adjusted for sleep, which is a significant factor in maternal return to activity (Davenport et al., 2025), and may be the primary driver of maternal MVPA and potential intermediary rather than partner’s domestic labor contributions. Qualitative investigation into how father’s domestic labor contributes to mother’s ability to return to PA may improve upon these results and provide more insight to the current study’s results.

Strengths of this investigation include the longitudinal design, application to a North American context, evaluation of perception between couples, capturing housework and childcare, and assessment of multiple health outcomes. The current investigation’s limitations pertain to the unassessed factors related to maternal and paternal health, generalizability, and modest sample size. First, relationship quality was not assessed in this study, although it is a potential intermediary variable through which domestic labor impacts maternal and paternal health (Biehle and Mickelson, 2012; deMontigny et al., 2020). The current investigation captured perception, which is another construct of couple agreement and incorporated into such models. Second, this study did not capture pharmacological assistance that may reduce parental stress, which may be utilized by both mother and father postpartum (Honkaniemi and Juarez, 2024). Third, the current study was conducted in Canada, which has extended leave for both mothers and fathers, and the sample was primarily White. Though many high-income countries have some form of leave and similar social norms for using leave and childcare distribution (Schindler et al., 2025), these results may not generalize to other countries where couples have less leave (e.g., the United States) (Cardenas et al., 2021; Schindler et al., 2025), or other race/ethnicities with differing domestic labor expectations and activity patterns postpartum (Zhang and Razza, 2022). The high utilization of leave also limited our investigation of parental leave on father domestic labor contribution, father stress, and parent’s health. Finally, this study had a modest sample size amongst couples (*n* = 52), and only 62 % of individuals provided complete data which may limit generalizability. Moreover, this study sought a convenience sample, and results may not generalize to all first-time parents. A larger and diverse sample of couples may improve upon testing power. Still, mixed models allowed for the use of additional data in the models, including individuals with missing couples’ data in the first aim.

Findings from this study provide implications for midwifery practice and for future research and theory for improved parental health. Maternity care implications include early conversations about fathers’ roles in housework and childcare during the postpartum period. This practice would align with the holistic focus and continuous nature of midwifery care, and acknowledging how social experiences may impact postpartum individuals (Midwives, 2025; Nurse-Midwives, 2025). Accordingly, emphasizing the benefits of mothers returning to PA and getting enough sleep should be part of these conversations. These practices may be refined by further research into three areas. First, exploration of father domestic labor in multiple child homes is needed to understand how domestic labor may change with subsequent children and support both parents throughout parenthood (Divine et al., 2021). Second, experimental evidence is needed to thoroughly test whether changing a father’s domestic labor in the postpartum period can have positive impacts on the parent’s health. The current study suggests that a parent-based approach, considering domestic labor, is well-positioned for success. However, further replication of these models in larger samples of intact couples using an actor-partner interdependence modelling may help address the effects of domestic labor on father’s own health and the spouse’s health. Finally, replication in other countries and with a larger number of couples may improve upon the current study’s results.

In this sample, fathers contributed a moderate amount of domestic labor throughout the postpartum period. Mother’s perception was positively related to fathers’ domestic labor. Fathers across domestic labor categories experienced higher stress at different times. Nonetheless, these early labor categories were related to mothers’ MVPA across the postpartum period. Interventional evidence is required to optimize domestic labor and new parent’s health.

## Supplementary Material

Supplementary Items

Supplementary material associated with this article can be found, in the online version, at doi:10.1016/j.midw.2026.104777.

## Figures and Tables

**Table 1:** Demographic characteristics of included participants (*n* = 164).

	Mothers (*n* = 88)	Fathers (*n* = 76)	Total
Age, Mean (SD)	30.7 (4.0)	32.5 (4.8)	31.5 (4.5)
Ethnicity, n (%)^#,^^			
White	75 (86.2)	60 (78.9)	135 (82.8)
Asian	9 (10.3)	9 (11.8)	18 (11.0)
Other	3 (3.4)	7 (9.2)	10 (6.1)
Education, n (%)^#^			
High school diploma	9 (10.2)	6 (8.0)	15 (9.2)
Vocational school or some college	10 (11.3)	4 (5.3)	14 (8.6)
College Degree	37 (42.0)	46 (61.3)	83 (50.9)
Professional or graduate degree	32 (36.3)	19 (25.3)	51 (31.2)
Current Breastfeeding, n (%)*			
2-months	83 (94.3)		
4-months	79 (90.8)		
6-months	79 (90.8)		
8-months	71 (82.5)		

# Missing 1 father report;.

^ Missing 1 mother report; Asian included individuals who identified as South Asian, Arab, Korean, Japanese, Chinese or Filipino; Other race included individuals who reported Other race, along with individuals who reported aboriginal or Latin American due to small numbers;.

* Variable only applies to mothers; missing 1 report at 4-months and 6-months, and 2 reports at 8-months.

**Table 2:** Stress and Physical activity based on father’s reported contribution to childcare and housework.

	2-month Estimate ± SE	4-month Estimate ± SE	6-month Estimate ± SE	8-month Estimate ± SE	Group	Time	Group*Time Interaction
*Father Stress*							
Low childcare and mixed housework	14.9 ± 4.1	13.0 ± 4.2	11.4 ± 4.0	12.1 ± 3.9	0.62	0.42	0.37
Moderate childcare and moderate housework	12.8 ± 2.5	14.5 ± 2.6	15.1 ± 2.5	14.8 ± 2.6			
Moderate childcare and high housework	12.9 ± 1.2	12.7 ± 1.3	12.1 ± 1.3	14.1 ± 1.3			
*Maternal Moderate-to-Vigorous Physical Activity (*min*/week)*							
Low childcare and mixed housework	101.7 ± 72.5	259.3 ± 78.3	222.1 ± 71.8	237.9 ± 71.6	0.04*	0.87	0.27
Moderate childcare and middle housework	416.0 ± 135.9	200.5 ± 132	163.7 ± 14.1	160.7 ± 122.1			
Moderate childcare and high housework	118.7 ± 39.0	170.3 ± 39.3	233.9 ± 40.2	244.7 ± 41.4			

^ Least squares means presented from individuals group models; resulted conducted using generalized linear mixed models with adjustment for time, maternity leave, nursing status, other parent’s perception of their housework contribution, other parent’s perception of their childcare contribution, and the parent’s own sleep;.

* *p* < 0.05.

## Data Availability

Data available on request from the authors.

## Statement of Significance

- **Problem or Issue:** The postpartum period is associated with poor maternal mental and physical health.
- **What is already known:** Efforts to support adequate parent health behaviors, and physical and mental health may have long-term benefits for the parents and child. Childcare and housework are opportunities for partners to support mothers in other life periods. Little is known about the postpartum period.
- **What this study adds:** This study documents, except for early postpartum, fathers contributed moderately to domestic labor postpartum. Opportunities to support fathers’ contribution to domestic labor in the postpartum period may help improve maternal health behaviors.
