# Supplementary material for: The association between paternal housework and childcare with parents’ health in the postpartum period
Source: Midwifery. Author manuscript; Available in PMC 2026 Apr 13. (PMC13075724; doi:10.1016/j.midw.2026.104777)
Supplement: Supplementary Items [file NIHMS2158560-supplement-Supplementary_Items.docx]

Panel A

Panel B

**Supplementary Figure 1. Distribution of Father’s Contributions across time (n=76)**

Panel A: Childcare Contributions; Panel B: Housework Contributions

| **Supplementary Table 1. Distribution of father-reported contributions to housework and childcare at 2-months** | | | | | |
| --- | --- | --- | --- | --- | --- |
|  | | | | | |
| ***Full sample (n=76)*** | | | | | |
|  | Housework category | | | Total |  |
| Childcare category | A little, 1-25% | some, 26-50% | A fair bit, 51-75% |  |  |
| ***Full sample (n=76)*** | | | | |  |
| A little, 1-25% | 1 (1%) | 5 (7%) | 0 | 6 (8%) |  |
| some, 26-50% | 15 (13%) | 15 (19%) | 1 (1%) | 29 (38%) |  |
| A fair bit, 51-75% | 6 (8%) | 29 (38%) | 6 (8%) | 41 (54%) |  |
| Total | 20 (26%) | 49 (64%) | 7 (9%) | 76 |  |
|  |  |  |  |  |  |
| ***Couple only sample (n=52)*** | | | | |  |
| A little, 1-25% | 1 (2%) | 3 (8%) | 0 | 4 (8%) |  |
| some, 26-50% | 8 (15%) | 11 (30%) | 1 (2%) | 20 (38%) |  |
| A fair bit, 51-75% | 1 (2%) | 22 (42%) | 5 (10%) | 28 (54%) |  |
| Total | 10 (19%) | 36 (69%) | 6 (11%) | 52 |  |
|  | | | | | |
